# Transcriptomic landscape of staminate catkins development during overwintering process in *Betula platyphylla*

**DOI:** 10.3389/fpls.2023.1249122

**Published:** 2024-01-08

**Authors:** Jingyun Zhang, Jiayuan Shi, Kehao Zeng, Mengjie Cai, Xingguo Lan

**Affiliations:** Key Laboratory of Saline-Alkali Vegetation Ecology Restoration, Ministry of Education, College of Life Sciences, Northeast Forestry University, Harbin, China

**Keywords:** *Betula platyphylla*, staminate catkins, microspore, transcriptome, weighted gene co-expression network analysis

## Abstract

*Betula platyphylla*, belonging to the cold-specialized lineage Betulaceae, exhibits a unique reproductive strategy where staminate catkins emerge in the first summer and undergo an overwintering process, culminating in flowering in the following year. However, the underlying regulatory mechanism remains unclear. In this study, we investigated the male germline development of *B. platyphylla* in four distinct stages: microsporocytes in Oct. (S1), uninuclear microspores from Dec. (S2) to Mar. of the following year (S3), and bicellular microspores in Apr. (S4). We performed RNA sequencing on mature pollen and the four stages of staminate catkins. Using weighted gene co-expression network analysis (WGCNA), we identified five highly correlated gene modules with distinct expression profiles. These modules exhibited strong correlations with sugar metabolism, cell cycle, flowering, and cell wall dynamics, highlighting their dynamic roles during male germline developmental stages. During the overwintering process, we observed that the expression of transcription factors such as *BpDUO1* and *BpAMS* at the appropriate developmental stages, suggests their significant roles in male germline development. The expression patterns of *BpFLC* and *BpFT* suggest their potential involvement in temperature perception during male reproductive development. These findings offer valuable insights into the reproductive success of plants adapting to cold environments.

## 1 Introduction

Angiosperm undergo a conserved male germline developmental process in the anther, where primary sporogenous cells differentiate into microsporocytes (Gómez et al., 2015). These microsporocytes then undergo meiosis to form tetrads, which eventually release four uninuclear microspores. Subsequently, the uninuclear microspores divide and develop into mature pollen grains consisting of a vegetative cell and two generative cells (Hafidh et al., 2016). The genetic regulation of these developmental processes is critical for successful male reproduction (Mandaokar et al., 2006; Wilson and Zhang, 2009). Many key genes, including *DUO1* (*DUO POLLEN 1*), *EMS1* (*EXCESS MICROSPOROCYTES1*), and *AMS* (*ABORTED MICROSPORES*), have been identified as essential regulators of male germline development (Zhao et al., 2002; Borg et al., 2011; Ferguson et al., 2017).

Cold stress often disrupts the male germline developmental process in cold-susceptible plants by decreasing reducing sugar availability and altering phytohormone levels, ultimately resulting in abnormal tapetal programmed cell death (PCD) and male sterility (Nayyar et al., 2005; Oliver et al., 2005; Oliver et al., 2007). In contrast, moderately low temperature induce the transition from vegetative to reproductive growth through a process called vernalization (Luo and He, 2020). Vernalization promotes the expression of flowing related genes, such as *FT* (*FLOWERING LOCUS T*), by downregulating the expression of *FLC* (*FLOWERING LOCUS C*), a key repressor of *FT* (Helliwell et al., 2006). The decline of *FLC* expression, mediated by epigenetic modification at the *FLC* locus, enables the transition from vegetative to reproductive growth in response to prolonged cold exposure (Bouché et al., 2017; Zhu et al., 2021). The dormancy phenomenon in trees shares similarities with vernalization (Chouard, 1960; Cooke et al., 2012).

Cold-tolerant plants have evolved adaptive strategies to cope with cold temperature during reproductive development (Campoy et al., 2011; Viti et al., 2013; Sharma and Nayyar, 2016). The genus *Betula* species, which is predominantly found in the northern hemisphere, exhibit staminate catkins that undergo a prolonged overwintering process and eventually achieve pollen dispersal (Chen et al., 1999; Wang et al., 2021). The reproductive processes in Betulaceae species have adapted to long-term low-temperature environments, including the slow development and dehydration of overwintering staminate catkins (Miller-Rushing and Primack, 2008). Climate factors, particularly air temperature, greatly influence the pollen concentration of birch (Jochner et al., 2013; Ranpal et al., 2022; Ranpal et al., 2023). Thus, it is essential to understand the molecular regulatory network underlying temperature response in this species for safeguarding long-term survival under climate change.

In this study, we investigated the developmental process of staminate catkins in *Betula platyphylla* trees during overwintering process. Throughout this process, male germline cells experienced a range of cold temperatures, from microsporocyte stages at chilling temperature to prolonged uninuclear microspore stages at freezing temperature, culminating in mature bicellular pollen. Weighted gene co-expression network analysis (WGCNA) of mature pollen and four stages of staminate catkins based on RNA sequencing (RNA-seq) revealed related biological processes during male germline development. Our results reveal that temperature fluctuation governs gene expression patterns of male germline development and pollen viability of *B. platyphylla* during the overwintering process.

## 2 Materials and methods

### 2.1 Plant materials

The staminate catkins and pollen were collected from outdoor *B. platyphylla* trees located at the Northeast Forestry University (45.7662°N, 126.6247°E). Staminate catkin samples were collected on Oct. 20 and Dec. 20, 2021, as well as on Mar. 20 and Apr. 20, 2022, respectively. Pollen samples were collected on May 1, 2022. Each sample was collected from three trees, and three biological replicates were used for each time point.

### 2.2 Sectioning and DAPI staining

The collected staminate catkins were fixed in FAA (Formalin-Aceto-Alcohol) with 70% ethanol: acetic acid: 37% formaldehyde = 18: 1: 1, for 48 hours, followed by five rounds of vacuuming for 1.5 hours each. The catkins were then dehydrated using a series of ethanol solutions with increasing concentrations: 50%, 70%, 85%, and 100%, with each concentration step lasting for 3.5 hours. Subsequently, the catkins were dehydrated using a series of xylene solutions with increasing concentrations: 50%, 70%, 85%, and 100%, with each concentration step lasting for 4 hours. The dehydrated material was gradually saturated with paraffin wax. The paraffin mass was sectioned into sections of 7 µm by ultramicrotome. The sections were then stained with 1% toluidine blue for visualization.

The staminate catkins were ground into powder to obtain germline cells. Germline cells were stained with 0.1 μg/mL DAPI (4’,6-diamidino-2-phenylindole) for 30 min and washed three times with PBS (phosphate buffered saline, pH = 7.4). The stained germline cells were observed using a DX51 fluorescence microscope (Olympus, Japan) under bright fields and ultraviolet illumination.

### 2.3 RNA extraction and cDNA library preparation

Samples were instantly frozen in liquid nitrogen and stored at -80°C. Total RNA was extracted using RNAprep Pure Plant Kit (DP441, Tiangen, China). The quality of the extracted RNA was assessed using a NanoPhotometer (Implen). The KAPA Stranded RNA-seq Kits (Roche) were employed for sequencing library preparation, and the library quality was evaluated using Agilent Bioanalyzer 2100 system (Agilent Technologies, CA, USA). Illumina RNA-Seq was performed by Metware Biotechnology Co. (Wuhan, China) using the Illumina Novaseq6000 system.

### 2.4 RNA-seq data profiling

The *Betula platyphylla* v1.1 reference genome was obtained from Phytozome (https://phytozome-next.jgi.doe.gov/info/Bplatyphylla_v1_1). Low-quality reads (Q20 ≤ 50%) were filtered out and Illumina adapters were removed using Cutadapt (Martin, 2011). The quality of the resulting clean reads was assessed using FastQC (Andrews, 2010).

Alignment of the clean reads to the reference genome was performed using Bowtie2 (Langmead and Salzberg, 2012). Gene counts were obtained using featureCounts, and fragments per kilobase of exon per million mapped reads (FPKM) values were calculated using the countToFPKM *R* package (Liao et al., 2014; Alhendi, 2019). Differentially expressed genes (DEGs) were identified using the DESeq2 *R* package with the following thresholds: adjusted *p*-value (*p*.adjust, FDR method) < 0.05 and absolute log_2_ fold change (|log_2_FC|) > 1 (Love et al., 2014).

Gene Ontology (GO) enrichment analysis was performed using the clusterProfiler *R* package (Yu et al., 2012). Gene functional annotations were based on the best match of BLAST searches against TAIR (https://www.arabidopsis.org/) and Swissprot protein databases (https://www.sib.swiss/swiss-prot) of genes. Weighted gene co-expression network analysis (WGCNA) was performed with soft thresholding power *β* = 8 using the WGCNA *R* package (Langfelder and Horvath, 2008). The similarity between genes (1-TOM) was calculated, and the genes with similar expression profiles were grouped into the same gene modules using hierarchical clustering and dynamic tree-cut, with a minimum size requirement of 30.

### 2.5 Quantitative real-time polymerase chain reaction

cDNA was synthesized with TransScript® One-Step gDNA Removal and cDNA Synthesis SuperMix (AT311, Transgen). The primers were listed in **Supplementary Table 1**. qRT-PCR was performed according to the protocol using TransStart® Top Green qPCR SuperMix (AQ131, Transgen), and three biological replicates were used for each experiment. The relative expressions of all tested candidate genes were normalized to the inner reference genes *BpTUB* (*BPChr11G09309*). The relative gene expression level was calculated by the 2^-ΔΔCT^ method.

## 3 Results

### 3.1 Phenotypes of staminate catkins and male germline development

**Figure 1:**
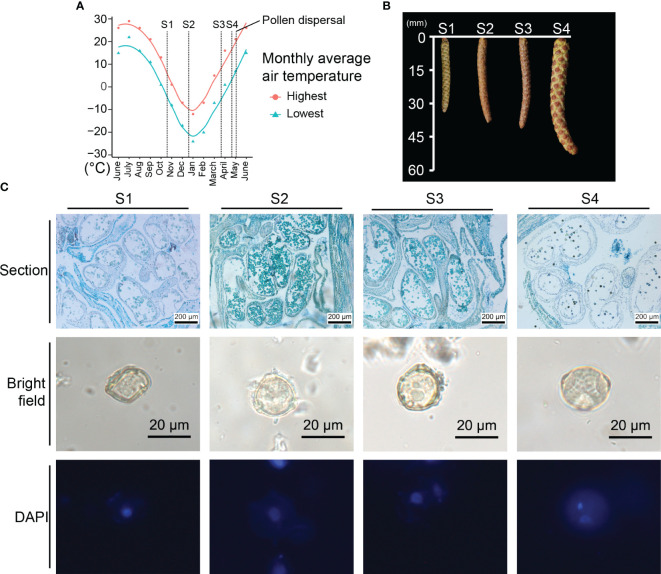
Phenotype and temperature condition of prolonged *Betula platyphylla* male development. **(A)** Monthly average temperature and development stages. y-axis: monthly average temperature of Harbin in °C from National Meteorological Information Center (http://data.cma.cn/). **(B)** Appearance and mean length of staminate catkins. **(C)** Phenotypic comparison of male germline stages.

The staminate catkins of *B. platyphylla*, which emerged in June 2021 and dispersed pollen in early May 2022, were subjected to a long period of low temperature during development. To investigate the staminate catkins development across winter, we selected four distinct stages. At S1, staminate catkins were subjected to chilling temperature (0-15°C) in the monthly average temperature from 1°C to 13°C. Staminate catkins at S2-S3 were subjected to freezing temperature (< 0°C) (**Figure 1A**). While at S4, the temperature increased to above zero temperature, and the mean length of staminate catkins increased from 34.2 mm at S1 to 59.6 mm at S4. During freezing temperature, staminate catkins at S2 and S3 exhibited a dehydrated state, which enhanced their freezing tolerance. As the temperature rises, staminate catkins at S4 underwent significant swelling in size and the anthers in catkins approached the pollen-releasing stage (**Figure 1B**; **Supplementary Table 2**).

We investigated the male germline development from *B. platyphylla* by performing sectioning and DAPI staining. The paraffin section and DAPI staining demonstrated a slow development across the four stages. Each anther of *B. platyphylla* often contains two locules. At S1, the locules contained interconnected microsporocytes. As the meiosis progresses from S1 to S2, uninuclear microspores differentiated and divided from microsporocytes, which significantly increased the cell numbers within the locules. From S2 to S3, uninuclear microspores with three pores were observed, and the phenotype had no significant changes during three months. At S4, the anther dehiscence occurred, and bicellular microspores were observed (**Figure 1C**).

Our findings suggest a microsporogenesis from S1 to S2, resulting in the formation of uninuclear microspores. From S2 to S3, uninuclear microspore development experiences a freeze-induced slowdown. Finally, at S4, the uninuclear microspores have developed into bicellular pollen, which is ready for release.

### 3.2 RNA-seq and differentially expressed genes analysis

RNA-seq was performed on pollen and staminate catkins from S1 to S4. The sequencing yielded a total of 1.2 billion high-quality clean reads. All the clean reads were aligned against the *B. platyphylla* reference genome (Chen et al., 2021), of which more than 91% were mapped and more than 82% were uniquely mapped, respectively (**Supplementary Table 3**). To obtain gene expression data, we calculated the fragments per kilobase of exon per kilobase million (FPKM) values of genes from the RNA-seq data for further analysis.

**Figure 2:**
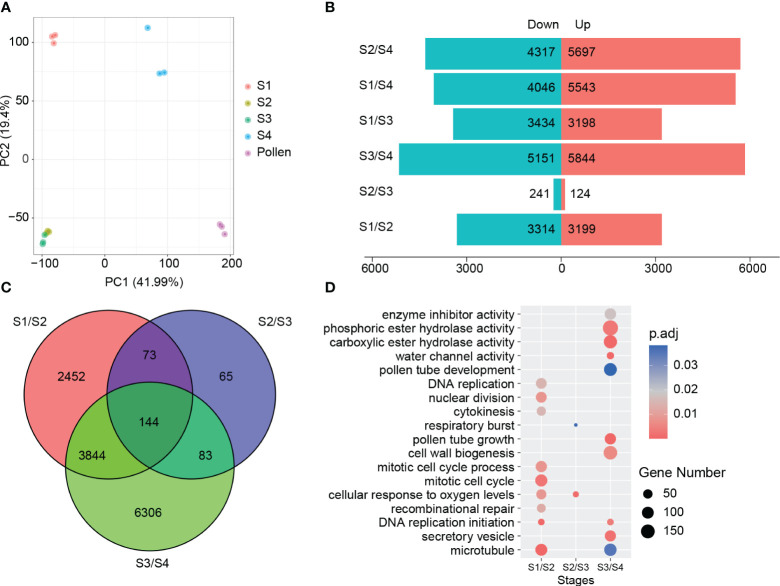
Overview of RNA-seq data. **(A)** PCA of 15 samples. **(B, C)** DEGs of four stages. **(D)** GO enrichment analysis of DEGs, gene counts representation in circular form.

Principal component analysis (PCA) using the gene expression data revealed a strong correlation within three replicates of each experimental group along PC1 (41.99%) and PC2 (19.4%). In contrast to the other experimental groups that could be clearly distinguished from each other, the S2 and S3 experimental groups showed significant overlap, suggesting limited differentiation between these two groups (**Figure 2A**).

We identified 6,513, 365, and 10,377 differentially expressed genes (DEGs) between adjacent stages in the comparisons of S2 vs S1, S3 vs S2, and S4 vs S3, respectively. A total of 12,967 DEGs were found to be differentially expressed in at least one of the examined periods. From S1 to S2, more DEGs (3,314) were down-regulated than up-regulated (3,199). In contrast, more DEGs were up-regulated (5,844) than down-regulated (5,151) from S3 to S4. Only 365 DEGs were found in the comparison of S3 vs S2, indicating a relatively smaller degree of regulatory changes in gene expression at freezing temperature (**Figures 2B, C**). In comparison of S4 and pollen, 4,392 DEGs were upregulated and 6,686 were downregulated (**Supplementary Table 4**). These findings exhibit the profound impact of substantial temperature fluctuations on male reproduction development, resulting in significant gene expression alteration.

GO enrichment analysis of the DEGs revealed the biological processes associated with different stages of male germline development in *B. platyphylla*. In the DEGs between S1 and S2, biological processes were enriched in “DNA replication” and “mitotic cell cycle”, suggesting activated cell division and growth during this process. On the other hand, the DEGs between S2 and S3 were found to be enriched in biological processes related to “cellular response to oxygen levels” and “respiratory burst”, suggesting a potential involvement of reactive oxygen species (ROS) in the response to prolonged cold temperature. Between S3 and S4, biological processes related to “cell wall biogenesis” and “water channel activity” were significantly enriched, indicating a focus on cellular structure and water transport in preparation for pollen release (**Figure 2D**). These findings suggested dynamic gene expression patterns and associated biological processes during male germline development in *B. platyphylla* during overwintering.

### 3.3 WGCNA analysis and key modules in temperature-related male germline development

**Figure 3:**
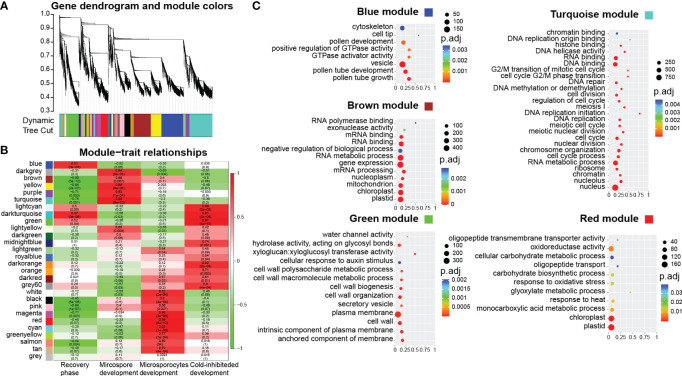
Co-expression network analysis. **(A)** Cluster dendrogram of different genes in co-expression modules. **(B)** Relationships between modules (left) and trait (bottom). **(C)** GO enrichment analysis of five modules. x-axis: rich factor.

To identify gene modules associated with male germline development in *B. platyphylla* from the four stages to mature pollen, we performed WGCNA to explore relationships between trait factors and gene co-expression modules. The trait matrix represented the developmental characterization in the five experiment groups (S1-S4 and pollen) using binary values. We defined four traits: recovery phase (“00011”) indicates the development process of S4 and pollen; microspore development (“01100”) indicates the development process of S2 and S3; microsporocyte development (“10000”) indicates the development process of S1; cold-inhibited development (“00010”) indicates the development process of S4, respectively. A total of 19,165 genes, filtered based on a mean FPKM value > 0.5, were grouped into 28 co-expression modules (**Figure 3A**; **Supplementary Figure 1**). We calculated pearson correlation coefficients and *p*-values for each module to assess their association with each trait. The modules significantly correlated with the four traits were identified.

**Figure 4:**
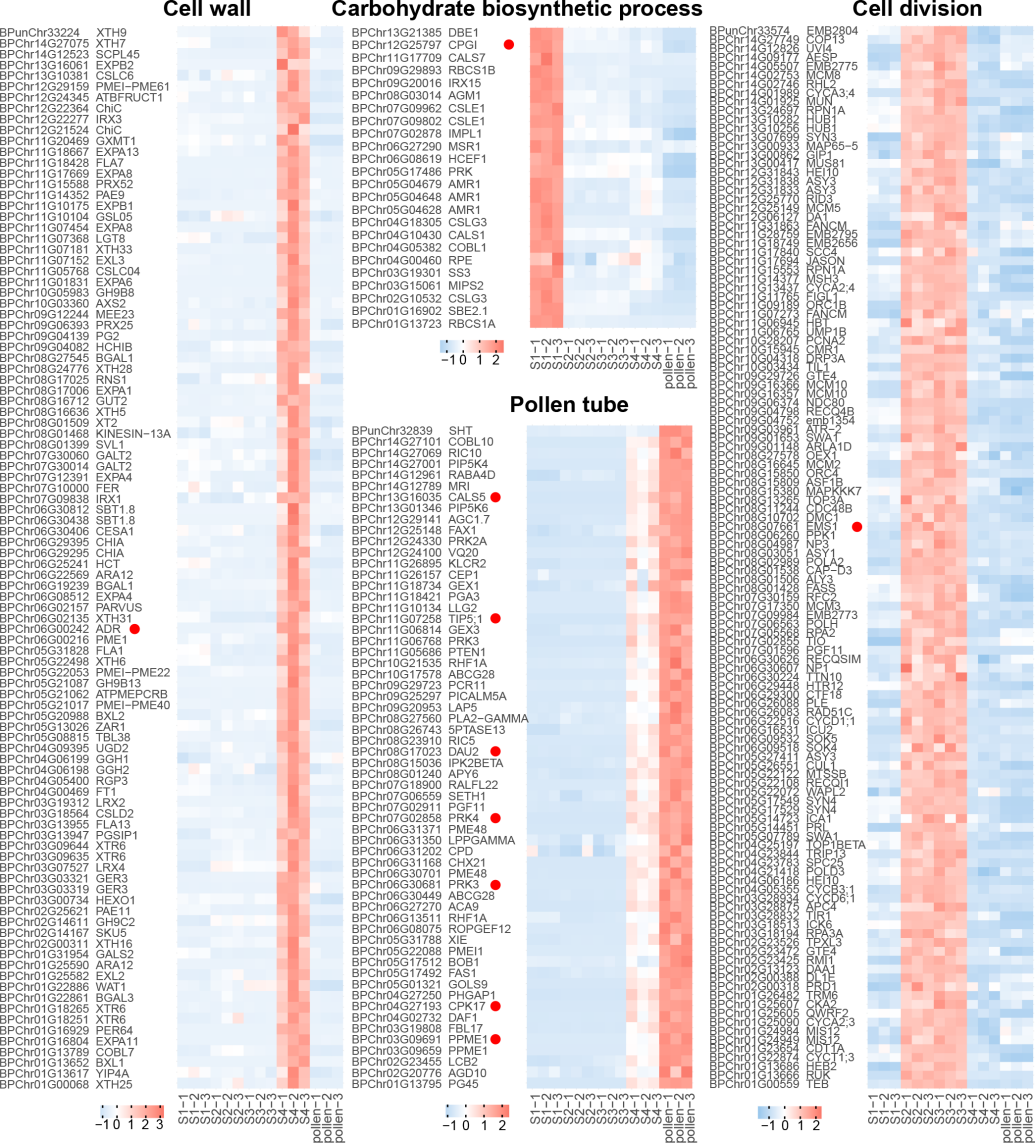
Gene expression of different biological processes. Each row represents a gene, and each column corresponds to different developmental stages (S1-S4 and pollen). The color scale indicates the standardized expression levels, with warmer colors representing higher expression and cooler colors indicating lower expression.

For the recovery phase, the blue module exhibited a significant positive correlation (*R* = 0.81, *p* < 0.001, **Figure 3B**; **Supplementary Figure 2**). GO enrichment on the genes in the blue module revealed that GO terms of “pollen tube” and “GTPase activity” were enriched (**Figure 3C**). As bicellular microspores emerge, pollen-specific genes such as *BpPRK3* (*BPChr06G30681*), *BpPRK4* (*BPChr07G02858*), *BpCPK17* (*BPChr04G27193*), and *BpPPME1* (*BPChr03G09691*) were upregulated at S4 and pollen (**Figure 4**). These genes play crucial roles in pollen tube growth (Tian et al., 2006; Myers et al., 2009; Takeuchi and Higashiyama, 2016). *CALS5* encodes a callose synthase involved in the synthesis and deposition of callose in the pollen exine wall (Dong et al., 2005). The low expression of *BpCALS5* (*BPChr13G16035*) from S1 to S3, followed by its upregulation at S4, suggested that pollen wall development was achieved by S4 (**Figure 4**).

On the other hand, the brown module exhibited a significant negative correlation with the recovery phase (*R* = -0.99, *p* < 0.001, **Figure 3B**; **Supplementary Figure 2**). This module showed significantly enriched in GO terms related to “gene expression”, “mRNA processing”, and “RNA metabolic process” (**Figure 3C**). This implies that cold temperature stimulates the upregulation of gene expression during male reproduction development.

For microspore development, the turquoise module exhibited a significant positive correlation under freezing temperature (*R* = 0.99, *p* < 0.001, **Figure 3B**; **Supplementary Figure 2**). GO enrichment analysis revealed that the terms associated with “cell division”, “DNA replication”, and “cell cycle G2/M phase transition” were significantly enriched within the turquoise module (**Figure 3C**). During overwintering, cell division emerges as a crucial metabolic process (Perry, 1971). The upregulation of *BpEMS1* (*BPChr08G07661*) at S2 and S3 highlights its essential role in microspore development (**Figure 4**). The *ems1* mutant exhibits male sterility and a lack of tapetal cells, indicating its essential function in microspore development (Zhao et al., 2002). The absence of modules significantly negatively correlated with microspore development suggests that cold temperature primarily regulated male germline development in an activating manner.

For microsporocyte development, the red module showed a positive correlation under chilling temperature (*R* = 0.97, *p* < 0.001, **Figure 3B**; **Supplementary Figure 2**). These genes belong to GO terms, including “cellular carbohydrate metabolic process” and “glyoxylate metabolic process” (**Figures 3C**, **4**). Among them, *BpCPGI* (*BPChr12G25797*), a cytosolic phosphoglucose isomerase was highly expressed at S1, whose homolog in *Arabidopsis thaliana* is essential for microsporogenesis (Liu et al., 2023) (**Figure 4**). These findings imply that sugar metabolism plays a crucial role in microsporogenesis under chilling temperature, and ensures sugar supply for subsequent microspore development.

For cold-inhibited development, the green module showed a strong positive correlation (*R* = 0.99, *p* < 0.001, **Figure 3B**; **Supplementary Figure 2**). The enrichment of GO terms within the green module, including “cell wall” and “water channel activity”, suggested their potential involvement in the late development of staminate catkins (**Figure 3C**). *ADR* (*ANTHER DEHISCENCE REPRESSOR*) acts as a key regulator of anther dehiscence by controlling ROS accumulation during secondary thickening in the anther cell wall (Dai et al., 2019). During the key stage of cell wall development, *BpADR* (*BPChr06G00242*) was upregulated specifically at S4 (**Figure 4**). When resource availability was limited, *B. platyphylla* might prioritize resource allocation towards crucial cellular processes necessary for microspore development and viability by suppressing specific genes and modifying expression patterns in response to low temperature.

Our findings suggest an active sugar metabolism under chilling temperature at S1, which meets the developmental requirements of the microsporocyte and subsequent microspore development. Under freezing temperature from S2 to S3, genes involved in cell division are upregulated, facilitating the development and preparation of uninuclear microspores for division. From S3 to S4, the expression of genes associated with cell wall metabolism and water transport is activated, enabling the successful development of uninuclear microspores into bicellular pollen.

### 3.4 Identification of hub transcription factors in WGCNA

**Figure 5:**
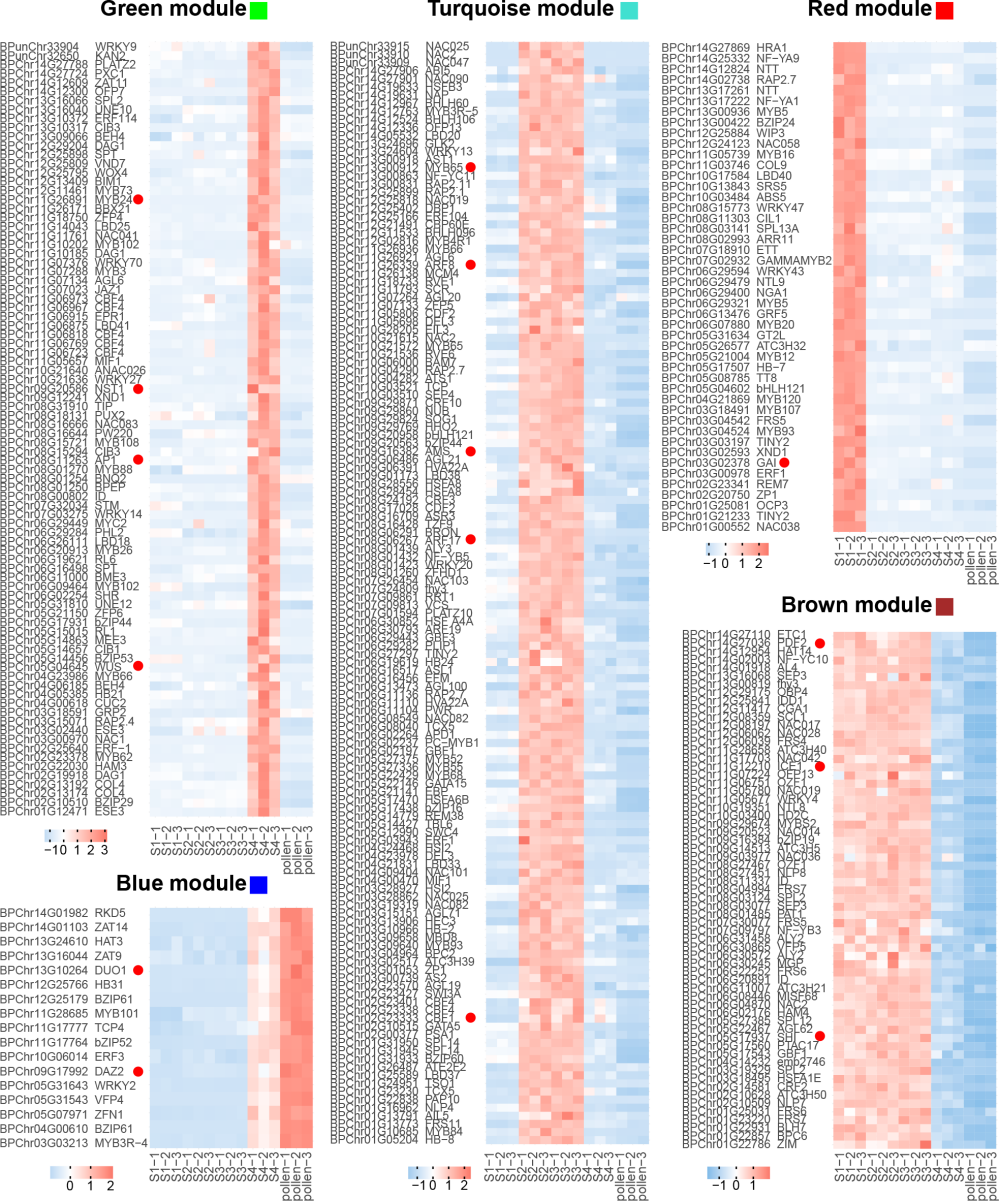
Gene expression of all hub TFs in five modules.

Hub genes, which serve as important regulators within the gene co-expression network, were identified based on thresholds with module membership > 0.8 and gene significance > 0.2. We extracted hub TFs, which may act as central regulators within the network. The blue module encompasses 17 hub TFs potentially involved in male germline development. We found that *BpDUO1* (*BPChr13G10264*) and its potential downstream targets, *BpDAZ2* (*BPChr09G17992*), *BpTIP5;1* (*BPChr11G07258*), and *BpDAU2* (*BPChr08G17023*) exhibited high expression at S4 and pollen, while their expression was low from S1 to S3 (**Figures 4**, **5**). *DUO1*, a member of the R2R3 MYB family, is reported to promote the cell division of uninuclear microspores (Borg et al., 2011). The expression pattern of *BpDUO1* explains the insufficient cell division of uninuclear microspores under the freezing temperature from S2 to S3.

In the brown module, hub TFs including *BpSHI* (*BPChr05G17937*) and *BpPDF2* (*BPChr14G27036*) suggest their essential involvement in the long-term development of staminate catkins from S1 to S3 (**Figure 5**). These findings align with the well-established roles of *SHI* (*SHORT INTERNODES*) and *PDF2* (*PROTODERMAL FACTOR 2*) in regulating stamen development (Ståldal et al., 2012; Kamata et al., 2013). We observed increased expression of *BpICE1* (*BPChr11G12210*) under cold temperature (**Figure 5**). *ICE1* (*INDUCER OF CBF EXPRESSION 1*) serves as an inducer of *CBFs* (*C-repeat binding factors*) and activates various processes related to cold tolerance and adaptation (Chinnusamy et al., 2007). *ICE1* is required for maintaining the dehydrated state of anthers, thereby enhancing cold tolerance during pollen development (Wei et al., 2018).

The hub TFs are specifically upregulated at S2 and S3 in the turquoise module (**Figure 5**). *BpAMS* (*BPChr09G16382*), whose homolog in *Arabidopsis* is known to be involved in anther and microspore development (Ferguson et al., 2017), exhibited specific upregulation under freezing temperature (**Figure 5**). Moreover, we observed an upregulation of *BpMYB65* (*BPChr13G00912*), *BpARF8* (*BPChr11G26339*), and *BpARF17* (*BPChr08G06267*) during uninuclear microspore development from S2 to S3 (**Figure 5**). These genes, *MYB65* (*MYB DOMAIN PROTEIN 65*) and *ARFs* (*AUXIN RESPONSE FACTORs*) are involved in gibberellic acid (GA)- and auxin-mediated anther development, respectively (Millar and Gubler, 2005; Wang et al., 2017; Ghelli et al., 2023). Furthermore, the increased expression of *BpCBF1* (*BPChr02G23333*) may enhance cold tolerance and the survival of *B. platyphylla* by regulating the response to cold temperature (**Figure 5**).

In the red module, *BpGAI* (*BPChr03G02378*), is upregulated under chilling temperature at S1 (Ito et al., 2018) (**Figure 5**). *GAI* (*GIBBERELLIC ACID INSENSITIVE*) encoding a DELLA protein, acts as a negative regulator of GA signaling and regulates microsporogenesis and anther dehiscence (Liu et al., 2017; Huang et al., 2022). The upregulation of *BpGAI* suggests a diminished GA signaling response under chilling temperature at S1. The subsequent downregulation of *BpGAI* from S2 to S4 suggests that the activation of the GA signaling pathway contributes to the adaption of microspore development to freezing temperature.

In the green module, flowering-associated hub TFs, including *BpAP1* (*BPChr08G11263*), *BpMYB24* (*BPChr11G26891*), and *BpWUS* (*BPChr05G04645*), showed a low expression pattern from S1 to S3, and a high expression level at S4 (**Figure 5**). These TFs have been previously characterized as crucial regulators of stamen development and male identity determination (Deyhle et al., 2007; Song et al., 2011; Huang et al., 2014; Wang et al., 2019). *NST1* (*NAC SECONDARY WALL THICKENING PROMOTING FACTOR1*) and *MYB108* are involved in anther dehiscence and pollen release (Mitsuda et al., 2005; Xu et al., 2019). *BpNST1* (*BPChr09G20586*) and *BpMYB108* (*BPChr08G15721*) showed significant upregulation at S4 (**Figure 5**), implying an activated cell wall metabolism occurred at this stage during anther development.

Our findings reveal the dynamic temperature-dependent regulation of microspore and anther development by GA and auxin responses. At chilling temperature, there is a low GA response at S1, which transitions to a high auxin and GA response from S2 to S3 under freezing temperature. Among the hub TFs, *BpAMS*, *BpMYB65*, *BpARF8*, and *BpARF17* may regulate microspore and anther development under freezing temperature from S2 to S3. The hub TFs involved in anther dehiscence and uninuclear microspore division show a low expression from S1 to S3 and a high expression at S4.

### 3.5 The expression profiles of genes related to vernalization pathway

**Figure 6:**
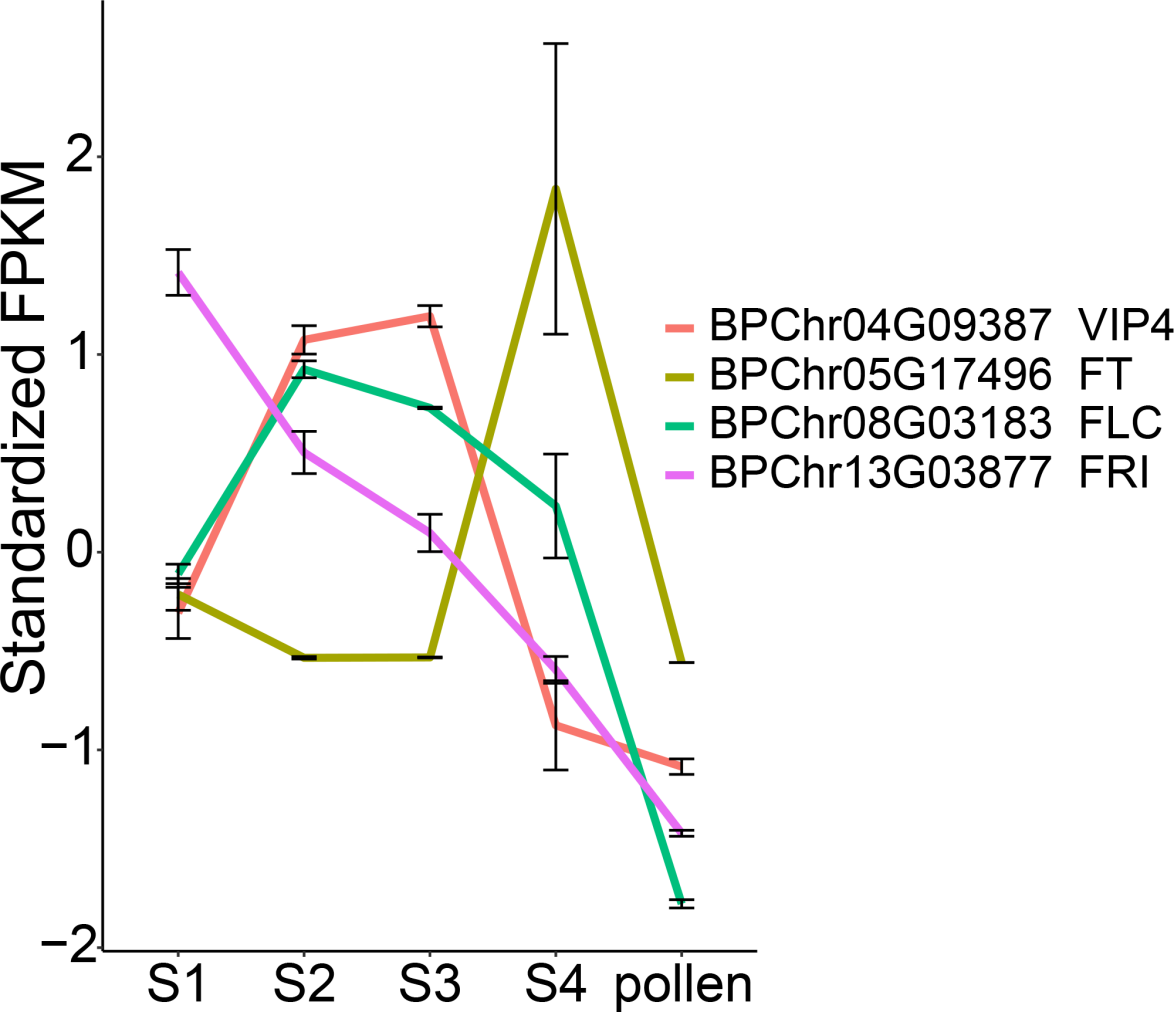
Gene expression of vernalization pathway. y-axis: standardized FPKM values.

Vernalization pathway play a crucial role in the inflorescence bud dormancy in addition to triggering the transition from vegetative to reproductive growth (Lu et al., 2022). We examined the expression of genes related to vernalization pathway. The expression level of *BpFLC* (*BPChr08G03183*), was induced by freezing temperature from S1 to S2, then gradually decreased from S2 to S4, along with *BpFRI* (*BPChr13G03877*). *FRI* (*FRIGIDA*) activates the transcription of *FLC*, and this activation was reported to be progressively repressed by epigenetic modification at the *FLC* locus (Li et al., 2018; Zhu et al., 2021). *VIP4* (*VERNALIZATION INDEPENDENCE 4*) was reported to positively regulate *FLC* expression, and its mutation renders the absence of *FLC* expression (Zhang and Van Nocker, 2002). *BpVIP4* (*BPChr04G09387*) exhibited high expression from S2 to S3. Specifically, *BpFT* (*BPChr05G17496*) showed high expression at S4, low expression at S1, and was undetectable at S2 and S3, indicating its repression under freezing temperature, possibly due to the high expression of its repressor, *BpFLC* (**Figure 6**). These observations suggest the involvement of vernalization pathway in the overwintering process of staminate catkins in *B. platyphylla*.

### 3.6 qRT-PCR validation of candicate genes

**Figure 7:**
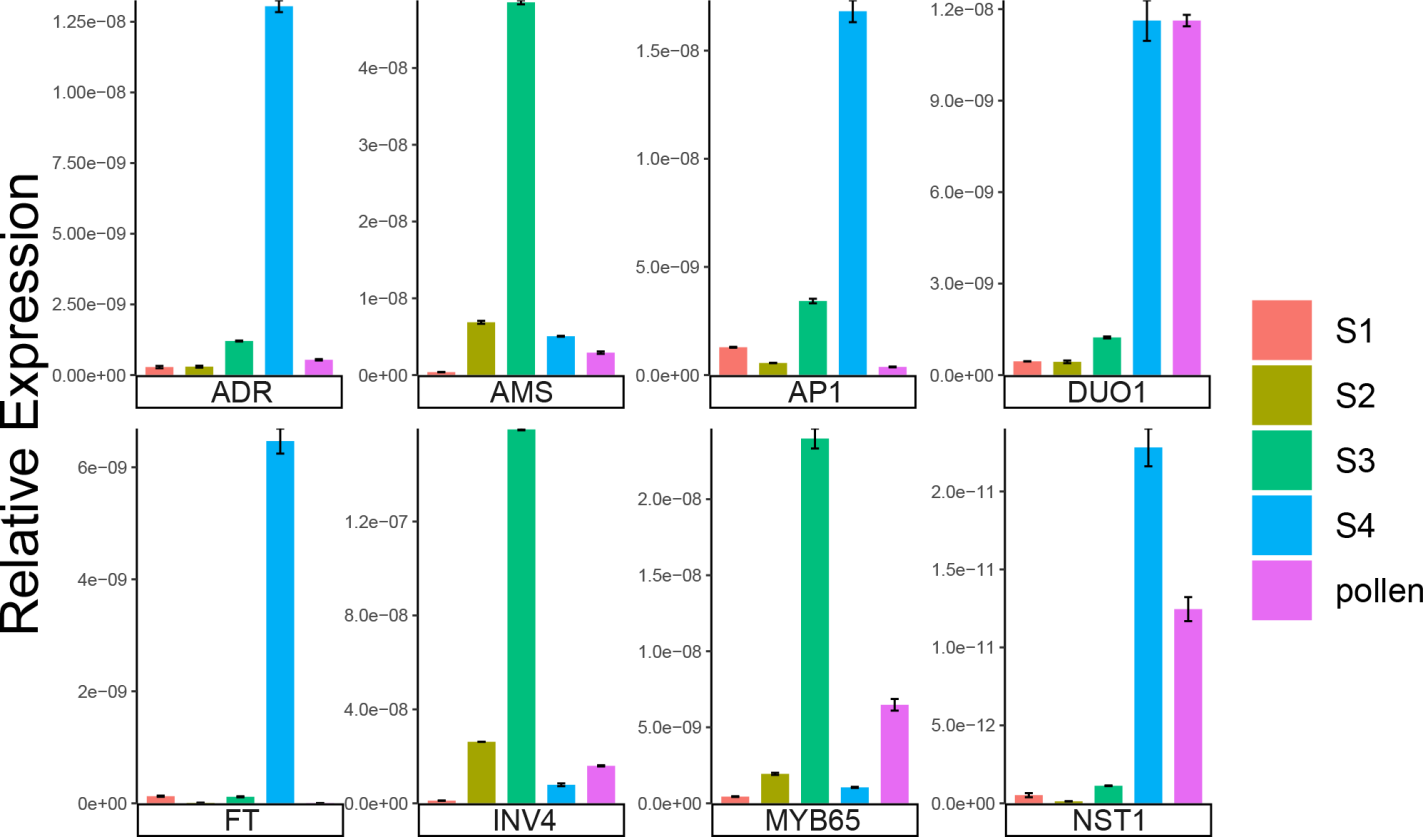
Relative expression levels of qRT-PCR. Error bars: standard errors.

We used qRT-PCR to verify the reliability of the RNA-seq data. A total of eight genes that may be involved in the development of staminate catkins in *B. platyphylla* during overwintering were selected. These genes included *BpADR*, *BpAMS*, *BpAP1*, *BpDUO1*, *BpFT*, *BpINV4*, *BpMYB65* and *BpNST1*. We found that the expression patterns of these genes obtained from qRT-PCR were consistent with the RNA-seq data (**Figure 7**; **Supplementary Figure 3**). The results revealed the reliability of the RNA-seq data.

## 4 Discussion

Trees, as perennial plants, have evolved various reproductive strategies such as flower dormancy to withstand prolonged cold exposure (Nilsson, 2022). Such phenomena have evolved distinct stages of male germline development in the face of different environments. Some plants undergo microsporogenesis after overwintering, e.g. *Prunus armeniaca*, while some occur before overwintering, e.g. *Rhododendron luteum* (Julian et al., 2011; Mirgorodskaya et al., 2015). Our observation of male germline development being arrested at the uninuclear microspore stage during overwintering in *B. platyphylla* indicates a unique adaptation strategy for reproductive processes in accordance with environmental conditions.

The overwintering process of trees involves intricate transcriptional regulation, including growth cessation, bud dormancy, cryoprotective process, and energy metabolism alteration (Chang et al., 2021). Similar to previous reports, the development of staminate catkins of *B. platyphylla* during winter may be involved in the regulation of *dehydrins* (*DHNs*), *FLC*, and *FT*, and energy metabolism related genes. Previous investigations have indicated that dehydrins expression in birch overwintering buds is temperature-responsive and may contributes to cryoprotection (Welling et al., 2004). In this study, the expression of *BpDHN1* (*BPChr05G17956*) in staminate catkins was upregulated at freezing temperature (**Supplementary Figure 4**).

Cold-tolerant plants employ diverse strategies to strengthen their resistance to low temperature, ensuring the proper development of male germline under cold stress (Sharma and Nayyar, 2016). In cold-susceptible plants, cold exposure typically leads to an increase in ABA levels, which inhibits PCD in the tapetum and leads to pollen abortion. *INV4* encodes a cell wall-localized invertase and is expressed in the tapetum. The expression of *INV4* is suppressed by a high level of ABA under cold exposure, which leads to pollen sterility by affecting the synthesis and accumulation of reducing sugars in the anthers. On the contrary, in cold-tolerant plants, cold stress upregulats the expression of ABA-8-hydroxylase genes (*ABA8ox1* and *ABA8ox2*), which are involved in decreasing ABA accumulation and improving reducing sugars (Oliver et al., 2005; Oliver et al., 2007; Ji et al., 2011). *BpINV4* (*BPChr05G08787*) and *BpABA8OX1* (*BPChr02G19591*) were upregulated during uninuclear microspore stages under freezing temperature, indicating the importance of low ABA content and increasing free reducing sugar content for microspore development under freezing temperature (**Supplementary Figures 3**, **5**). Additionally, our analysis indicates the potential involvement of related biological processes in cold adaptation, such as GA and auxin signaling pathways, and anther dehydration. The enrichment of GO terms associated with microsporocyte development before overwintering, including sugar biosynthesis and glyoxylate metabolism, supporting the resource storage hypothesis (Bogdziewicz et al., 2020). These metabolic adaptations in cold-tolerant species provide the necessary regulatory mechanisms for successful male germline development under cold temperature.

Temperature plays a significant role in the reproduction and development of trees, influencing the dormancy cycles and fulfilling the developmental requirements of overwintering flowers (Perry, 1971; Campoy et al., 2011; Viti et al., 2013). Previous studies have reported a negative correlation between temperature and birch pollen production (Jochner et al., 2013). Specifically, elevated temperature correlated with the upregulation of key genes such as *BpDUO1*, *BpADR*, *BpNST1*, and *BpMYB108*. These genes may be associated with uninuclear microspore division and anther dehiscence, thereby facilitating the effective release of pollen. Furthermore, the exclusive upregulation of potential key genes, such as *BpEMS1*, *BpAMS*, and *BpMYB65*, are involved in uninuclear microspore development under freezing temperature, suggests that insufficient cold accumulation may result in partial pollen sterility. These findings suggest a hypothetical gene regulatory pattern of the temperature fluctuation on pollen viability in *B. platyphylla*.

## 5 Conclusion

Our study provides a comprehensive transcriptomic analysis of staminate catkin and pollen development in *Betula platyphylla* under specific temperature fluctuation conditions. Our findings offer valuable insights into the molecular networks governing cold tolerance, male germline development, hormone signaling, flowering control, and the vernalization pathway. This research contributes to a better understanding of the adaptive traits in *B. platyphylla* and serves as a valuable resource for future studies in tree breeding and ecological management.

## Data availability statement

The data presented in the study are deposited in the NCBI Sequence Read Archive (SRA) repository, accession number PRJNA994611.

## Author contributions

JZ, JS, and XL conceived the study and research plans. JZ and MC collected plant materials and performed the experiments. JS and KZ analyzed the data. JS organized figures and tables. JS drafted the manuscript. XL revised the manuscript. All authors contributed to the article and approved the submitted version.

## Supplementary material

Supplementary Figure 1 Gene number of each module.

Supplementary Figure 2 The correlation between gene significance and module membership in the five modules.

Supplementary Figure 3 Integrative Genomics Viewer tracks displaying of gene expression coverage.

Supplementary Figure 4 Gene expression of *BpDHN1*.

Supplementary Figure 5 Gene expression of *BpINV4* and *BpABA8OX1*.

Supplementary Table 1 Primer of qRT-PCR.

Supplementary Table 2 Sampling time and mean length of staminate catkins.

Supplementary Table 3 RNA-seq and alignment summary statistics.

Supplementary Table 4 DEG data of RNA-seq.
