# Supplementary material for: Transcriptomic landscape of staminate catkins development during overwintering process in Betula platyphylla
Source: Front Plant Sci. 2024 Jan 8;14:1249122. doi: 10.3389/fpls.2023.1249122 (PMC10801112; doi:10.3389/fpls.2023.1249122)
Supplement: Supplementary Figure 1 — Gene number of each module. [file DataSheet_1.docx]

Supplementary Material

Gene Co-expression Network Analysis of Cold-Stressed Pollen Development in *Betula platyphylla* Staminate Catkins

Jingyun Zhang*, Jiayuan Shi*^­­^

*** Correspondence:** Xingguo Lan: lanxingguo@nefu.edu.cn

# Supplementary Figures and Tables

## Supplementary Figures


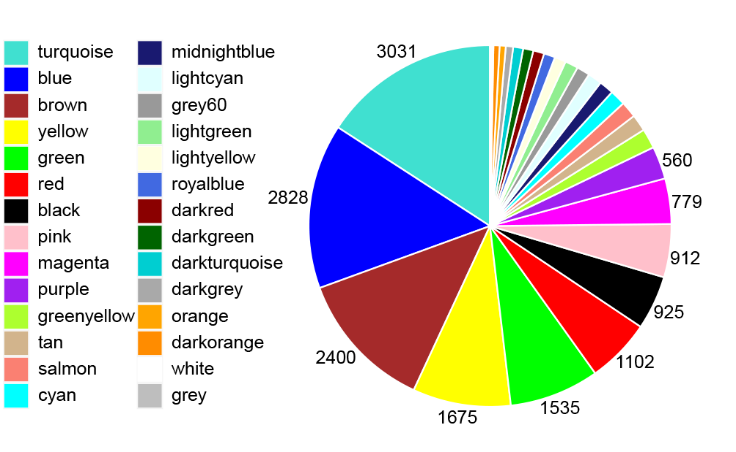


**Supplementary Figure 1.** Gene number of each module.


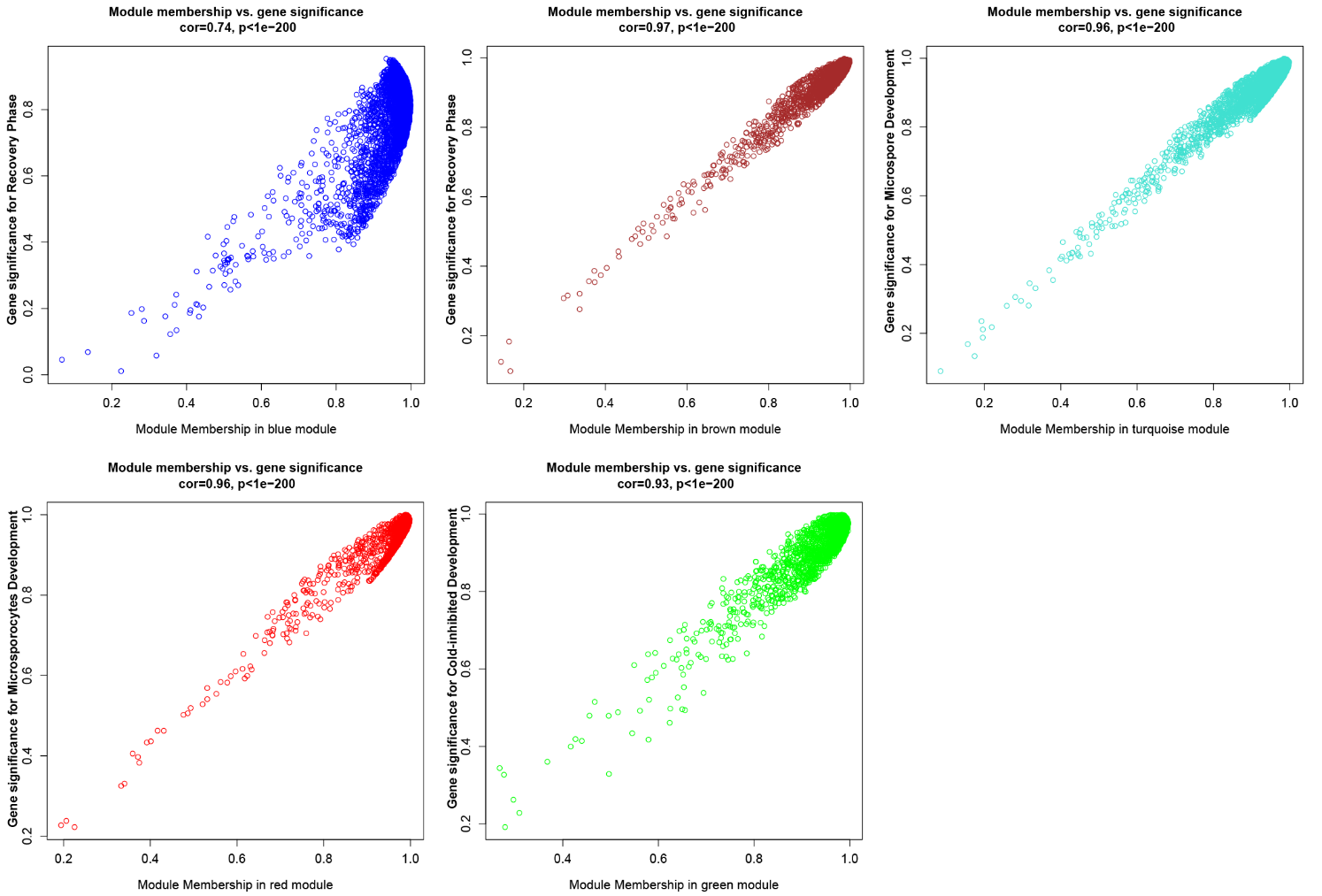


**Supplementary Figure 2.** The correlation between gene significance and module membership in the 5 modules.


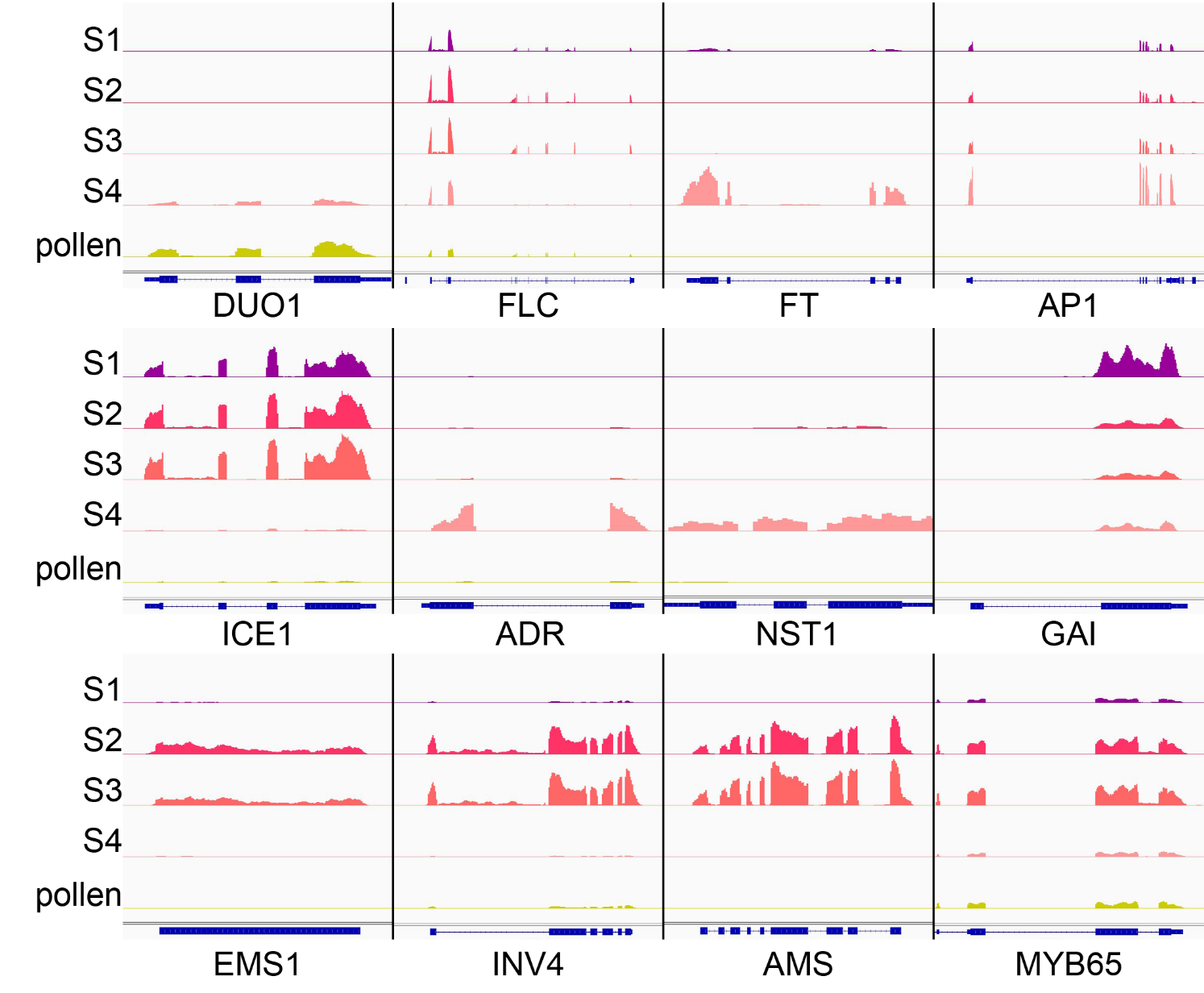


**Supplementary Figure 3.** Integrative Genomics Viewer tracks displaying of gene coverage.

**
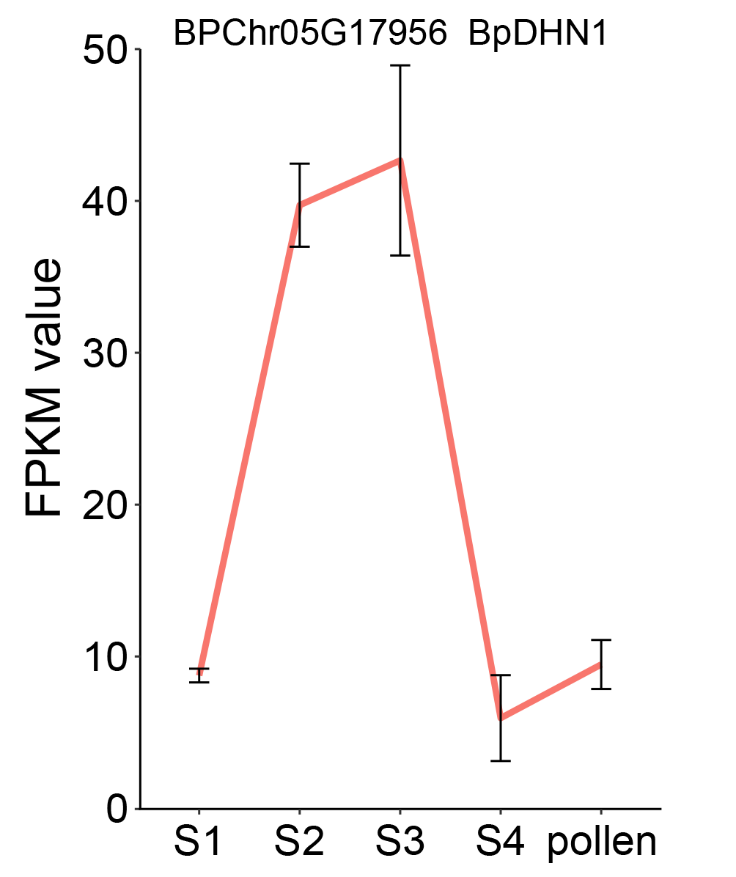
**

**Supplementary Figure 4.** Gene expression of *BpDHN1*.


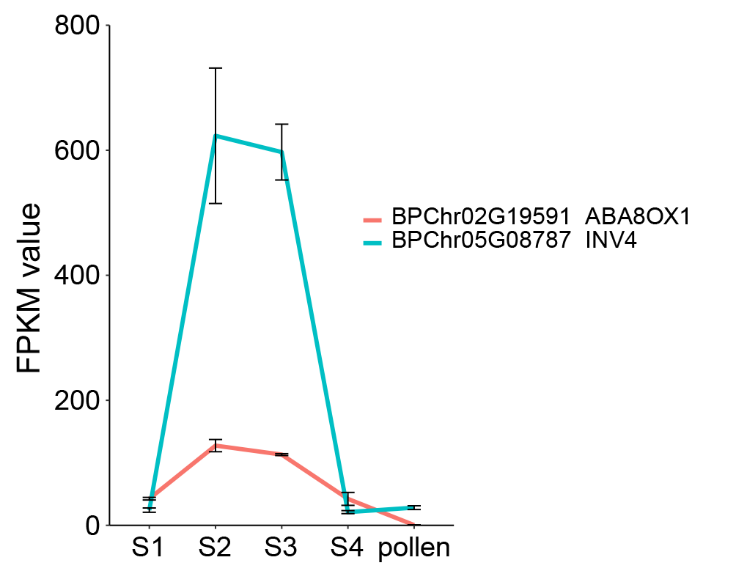


**Supplementary Figure 5.** Gene expression of *BpINV4* and *BpABA8OX1*.

## Supplementary Tables

| Name | Gene ID | Primer sequence (5’-3’) |
| --- | --- | --- |
| BpDUO1-F | BPChr13G10264 | GGACAGTACCCTCCAAATCACA |
| BpDUO1-R |  | CCTCCGACGAAGAACTGAACT |
| BpADR-F | BPChr06G00242 | TGTGGAGAAATGGAAGCAGCTA |
| BpADR-R |  | GCCAGCCACTTCTTTCTGTAAC |
| BpINV4-F | BPChr05G08787 | GGTGTTGGTCTGGCTCTA |
| BpINV4-R |  | AAGGCACTCGCATTCATC |
| BpAMS-F | BPChr09G16382 | TGAACTCTCTGGGACTGGAAGT |
| BpAMS-R |  | TCGCGTTAGCTCAAGCAAGG |
| BpMYB65-F | BPChr13G00912 | GGCAAGGATGGCAGCACAT |
| BpMYB65-R |  | TCAGGCGGATAAAGAGGTAAGC |
| BpAP1-F | BPChr08G11263 | CAATACAGGAGCAGAATACC |
| BpAP1-R |  | TAGCAGAAACGACGATGA |
| BpNST1-F | BPChr09G20586 | TCGCTGATGAGAAGATTG |
| BpNST1-R |  | TGAAGCCGCTGTAGATGA |
| BpFT-F | BPChr05G17496 | GCGATGATCTCAGGACCTTCTA |
| BpFT-R |  | CGCCTGTAGTTGCTGGAATATC |
| BpTUB-F | BPChr14G12622 | GACATCTGCTTCCGTACTCTGA |
| BpTUB-R |  | GGTACTGCTGTGATCCTCTTGA |

**Supplementary Table 1.** Primer of qRT-PCR.

| Samples | Sampling time | Mean length of staminate catkins (mm) | |
| --- | --- | --- | --- |
| S1 | October 20, 2021 | | 34.2 |
| S2 | December 20, 2021 | | 39.1 |
| S3 | March 20, 2022 | | 42.5 |
| S4 | April 20, 2022 | | 59.6 |
| Pollen | May 1, 2022 | | —— |

**Supplementary Table 2.** Sampling time and mean length of staminate catkins

| Sample | Raw Reads | Clean reads | Q20(%) | GC Content(%) | Reads mapped | Unique mapped |
| --- | --- | --- | --- | --- | --- | --- |
| S1-1 | 60711422 | 58326140 | 97.38 | 46.7 | 53390160(91.54%) | 48624460(83.37%) |
| S1-2 | 67845952 | 64743240 | 97.41 | 46.89 | 59099798(91.28%) | 53814445(83.12%) |
| S1-3 | 67817048 | 62464444 | 97.48 | 46.73 | 56997837(91.25%) | 51822439(82.96%) |
| S2-1 | 65131560 | 62669978 | 97.33 | 46.29 | 57539511(91.81%) | 52477517(83.74%) |
| S2-2 | 92084572 | 89198400 | 97.3 | 46.22 | 81845481(91.76%) | 74536755(83.56%) |
| S2-3 | 80258276 | 78466946 | 97.39 | 46.19 | 72003479(91.76%) | 65604613(83.61%) |
| S3-1 | 86058922 | 83467580 | 97.34 | 45.99 | 76565686(91.73%) | 69836712(83.67%) |
| S3-2 | 95326676 | 91198198 | 97.41 | 46.09 | 83959192(92.06%) | 76689954(84.09%) |
| S3-3 | 91084544 | 87091840 | 97.37 | 45.86 | 79693689(91.51%) | 72771746(83.56%) |
| S4-1 | 75325310 | 72418092 | 97.34 | 46.7 | 66453695(91.76%) | 60217052(83.15%) |
| S4-2 | 71800094 | 68168912 | 97.34 | 47.17 | 63072017(92.52%) | 57102928(83.77%) |
| S4-3 | 57551718 | 56446096 | 97.27 | 47.85 | 47525737(84.20%) | 42986174(76.15%) |
| Pollen-1 | 118908282 | 113952246 | 97.34 | 46.23 | 104868741(92.03%) | 93569427(82.11%) |
| Pollen-2 | 115030648 | 109381160 | 97.28 | 46.16 | 99923962(91.35%) | 88860607(81.24%) |
| Pollen-3 | 114168848 | 109334666 | 97.34 | 46.42 | 100651184(92.06%) | 89703806(82.05%) |

**Supplementary Table 3.** RNA-seq and alignment summary statistics.
